# Risk factors for type 2 diabetes mellitus among out-patients in Ho, the Volta regional capital of Ghana: a case–control study

**DOI:** 10.1186/s13104-017-2648-z

**Published:** 2017-07-26

**Authors:** Horlali Yao Gudjinu, Bismark Sarfo

**Affiliations:** 1 Jasikan District Health Directorate, P. O. Box 83, Jasikan, Volta Region, Ghana; 2 Department of Epidemiology, School of Public Health, University of Ghana, Legon, Accra, Ghana

**Keywords:** Diabetes mellitus, Risk factors, Out-patients department

## Abstract

**Background:**

The prevalence of type 2 diabetes mellitus in developing countries like Ghana continues to rise. This study seeks to assess the risk factors of type 2 diabetes mellitus in a Ghanaian setting. An unmatched case–control study among patients receiving care at the out-patient departments of the two major hospitals in the Ho Municipality. Patients diagnosed with type 2 diabetes mellitus were recruited. Appropriate controls with similar ages who were also patients receiving care at the out-patient department of these hospitals were recruited. Both cases and controls were administered a questionnaire that comprises of standardized and validated tools. These tools include WHO STEPs instrument, general practice physical activity questionnaire and rapid eating and activity assessment for patients. Additionally, the research participants were made to undergo physical examinations for weight, height, waist circumference and laboratory testing of fasting venous blood to assess the biochemical factors of interest namely fasting blood glucose and fasting lipids. Analysis of data was done using STATA version 11.

**Results:**

A total of 136 (48 cases and 88 controls) participants of which 95 [39 (81.25%) cases and 56 (63.64%) controls] respondents underwent laboratory testing for fasting blood glucose and fasting blood lipid (total cholesterol, HDL cholesterol and triglycerides). Participants were aged between 35 and 62 years. This study reveals a number of risk factors for type 2 diabetes mellitus. Individuals in the middle socio-economic class have a greater risk of developing type 2 diabetes mellitus with an OR of 5.03 (p < 0.003; 95% CI 1.71–14.74). Eating large quantities/servings of fruits per seating provides protection against development of type 2 diabetes mellitus. A low physical activity level is a valid determinant of type 2 diabetes mellitus irrespective of body mass index, socio-economic level or place of residence.

**Conclusions:**

Individuals within the middle socio-economic level, who are physically inactive and do not consume large amounts of fruit are at greatest risk of developing type 2 diabetes mellitus. Living in a rural setting is attendant with high levels of physical activity this tends to protect rural residents from type 2 diabetes mellitus. Physical activity level confounds the relationship between place of residence and development of type 2 diabetes mellitus. Local policies should be realigned to attract individual of the middle socio-economic level to live in rural areas where they are more likely to be both physically active and consume more fruits thus averting the risks of developing T2DM.

## Background

Diabetes mellitus (DM) is not a single disease but rather a group of diseases with types and a varied range of attributable causes. They are generally chronic in nature and characterized by persistently elevated blood glucose resulting from lack of sufficient insulin and or resistance to the action of insulin in the body [1, 2]. Some types of diabetes mellitus include; type 1 diabetes mellitus (T1DM—formally referred to as ellitus (T2DM—formally referred to as non-insulin dependent diabetes mellitus); latent autoimmune diabetes of adults (LADA); maturity onset diabetes of the young (MODY) and gestational diabetes [3].

Several risk factors have been identified for T2DM by various researchers across the globe. These risk factors fall under the four broad groups of risk factors for non-communicable diseases (NCDs). A good number of these risk factors border on life style and diet [4–7]. Another way of considering the risk factors of the T2DM is from a preventive, treatment or an interventional point of view which looks at the various risk factors as modifiable or non-modifiable [8]. This way, modifiable risk factors like obesity, harmful use of alcohol, physical inactivity and dietary factors can be altered in the prevention of the disease. Non-modifiable risk factors like age, ethnicity and family history may serve to alert an individual of his or her baseline risk of contracting the disease. The risk of an individual developing T2DM is dependent on the total presence and combination of exposure to both modifiable and non-modifiable risk factors.

Diabetes mellitus is a big complex worldwide health challenge in the 21st century. Although this problem has been recognised, many of the world’s public health experts and governing bodies remain blind to the real and current magnitude and scope of this problem [9]. This is particularly true in Africa and the developing world as a whole. Most importantly the implications of this threat to the future of health in our countries are receiving minimal attention. It was estimated that in the year 2004 alone 3.4 million individuals died from diabetes mellitus or of its complications and over 80% of these deaths occurred in middle or low income countries of which Ghana is one [10].

The developing world is the most affected by diabetes and at the same time it is the region with minimal amount of data or studies conducted to investigate this health problem. Over three (3.5) million people in the developing world died of diabetes mellitus in the year 2007 and 6 million new cases were diagnosed in the same year [11]. We are obviously in an atypical epidemiological transition resulting in a double burden of diseases both communicable and non-communicable but we are saddled so much by the communicable burden that we leave the NCDs to worsen [9, 11, 12]. Other studies found the prevalence to be 6.3% in Accra and concluded that the predictors of diabetes in Ghanaians must be the focus of future studies [4, 13].

There are marked discrepancies in the African region for the prevalence of diabetes. The prevalence varies by ethnicity, age, community, level of obesity (both population and individual) and composition of diet. While the estimated prevalence for the continent is 3.2% for adults, there is a 5:1 ratio of prevalence between urban and rural dwellers in studies carried out in Tanzania [9]. The effects of diabetes mellitus on the individual, community or nation are enormous but insidious. The low and middle income countries are the poorest in the world and expenditure on health is least in these countries but now they will be forced to spend their meagre earnings treating NCDs [12, 14].

The earliest available published research work on diabetes mellitus in Ghana recognized a prevalence of 0.2% in Ho, Volta Region among men in 1964 [15]. Poorly planned urbanization with the corresponding changes in life style and behaviour as well as globalization [10, 16] typically in the developing countries is the major fuel for NCDs. A community based survey report in the Greater Accra Region of Ghana showed the prevalence of diabetes to be 6.4% among adult Ghanaians aged 25 and above [13]. The prevalence of 0.2% in 1964 is far lower than 6.3%. Even though these are two different cities the difference is still alarming. Furthermore, a study in Kumasi found the prevalence to be 9.26% among Pentecostal Christians [17].

The aim of this study was to assess the risk factors of T2DM among a section of hospital clients in Ho. The Ho Municipal hospital in 2010 attended to 3556 diabetic patients at the out patient department (OPD). This number however rose to 10,034 and 9876 in 2011 and 2012 respectively according to unpublished data from the dhims2 of the Ghana health services. It is not only the disease prevalence that is rising in the country but correspondingly, the major risk factors for diabetes are also on the increase. Obesity for instance is 10% among men and 36% among women who are civil servants in Accra [18]. It is clearly evident that diabetes mellitus with its attendant risk factors are gradually becoming a major health concern in Ghana that requires scientific studies.

### Objectives

The specific objectives of the study was to evaluate the risk factors for T2DM along the lines of socio-demographic, anthropometric and physical activity factors; dietary factors and biochemical factors among general population of patients who visit the OPD within the Ho municipality.

## Methods

### Study design

This study was an unmatched case–control study. In this study, patients diagnosed with T2DM receiving care at the two government hospitals of the Ho Municipality were recruited. Appropriate controls with similar ages who are also patients (but non-diabetic patients) receiving care (for conditions such as malaria, respiratory tract infections, gastroenteritis, anaemia, enteric fever, hypertension, intestinal worm infestations etc.) at these hospitals were recruited alongside with them. Both cases and controls were administered a questionnaire that comprises of standardized and validated tools. These tools include WHO STEPs instrument, GPPAQ and REAP. Additionally, the research participants were made to undergo physical examinations for weight, height and waist circumference. The participants were made to undergo laboratory testing of fasting venous blood to assess the biochemical factors of interest namely fasting blood glucose and fasting lipids. The distinction between those with the disease and those without the disease was based on the WHO criteria (for case definition for diabetes) [19]. Diabetes patients on lipid lowering drugs were excluded. Comparison of cases to controls for the various risk factors under different groupings namely; socio-economic status (SES), demographic and physical activity; dietary factors and biochemical risk factors was made. To be eligible for recruitment, the client must be living within the Ho Municipality; be within the ages of 35–65 years; must not be severely sick or bed ridden but be an otherwise normal ambulant out-patient. The diagnostic criteria for T2DM were as follows: Any patient aged at least 35 years but not older than 65 years with FBG of ≥7.0 mmol/L or a known diabetes mellitus patient diagnosed after the age of 35 years on oral anti-diabetic treatment. Any patient receiving care at the OPD of these two hospitals: Who is not a known diabetic and had laboratory confirmation of fasting blood glucose to be <7.0 mmol/L was recruited as control.

### Study setting

Ho is the capital of the Ho Municipality located in the heart of the Volta Region of Ghana. It is also the regional capital. The Republic of Togo is on the Eastern border of the Ho Municipality and in the southern border is the Adaklu-Anyigbe District, in the western border is the South-Dayi district and the Hohoe Municipality is located on the northern border. The people of Ho are of the Ewe tribe however given its status as regional capital and municipal capital, it has attracted various groups of people from all over Ghana and beyond. It is therefore becoming a heterogeneous society.

There are two diabetic clinics in the Ho Municipality which provide care to the diabetic patients. These two clinics are also housed in the two major hospitals of the municipality that provide care to most patients in the municipality. The study population for this research was patients receiving clinical care at these two hospitals. Only otherwise, able and ambulant patients receiving care on out-patient basis at these two hospitals were recruited. In-patients on the hospital wards were not recruited as subjects in this study. The study used both incident and prevalent cases of T2DM. Both the cases and controls were patients receiving care at these two hospitals. This study was conducted in the Ho Municipality during the months of May to July, 2014.

The estimated prevalence of diabetes for Ghana is 3.35% [18] and the population size of Ho Municipal was 169,669 in 2010 according to unpublished data in the dhims of the Ghana Health Services. The highest reported prevalence of diabetes was in Kumasi among Pentecostal Christians which stood at 9.26% [17]. A similar case–control study among diabetes estimated the proportion of obesity among diabetics to be 58% whiles among the controls it was 33% [6].These values were used in the Epi-Info 7 software. Using these values with a power of 80%, confidence level of 95% and a ratio of 1:2 for cases and controls, a sample size of 46 cases with 91 controls (Total 137—Fleiss formula/method) is required. A total of 136 subjects were recruited for this study with 48 cases and 88 controls.

### Data collection

Data on the cases and controls was collected using different tools merged together into one questionnaire. The main core of this instrument is the WHO STEPwise approach to surveillance (STEPS) which is a standardized tool design purposefully to be employed in the surveillance for risk factors for NCDs in member countries [20]. For the purposes of this study and easier analysis, some components of the STEPS will not be applicable and other segments will be too extensive so those parts were not included. Two additional standardized tools were incorporated. These were, a physical activity level assessment tool which is in the form of a questionnaire that is administered and it generates four categories of outcome. It is known as the general practice physical activity questionnaire (GPPAQ). It was developed by the London school of hygiene and tropical medicine [21]. The second is a more detailed dietary survey instrument. It is called the rapid eating and activity assessment for patients (REAP) developed for use in clinical settings [22].

Diagnosis of diabetes status was based on fasting plasma glucose levels as tested in the laboratory. Blood pressure measurement was done three times or as checked on three different occasions and the most consistent readings for systolic and diastolic blood pressures were taken as the true values. Information/data obtained from the patient interviews and the various data collection tools was cross checked with what is available on the patient’s hospital records, folder or insurance card whenever necessary and possible to ensure consistency.

### Statistical analysis

Data obtained from this study was first examined for completeness and consistency as part of quality control check measures after which they were compiled into a spread-sheet format using the EpiData version 3.1 software which was subsequently exported into the Stata version 11 (StataCorp, College Station, TX, USA) for statistical analysis. Where missing data was encountered during the process of compiling the data, the client hospital records were retrieved to cross check. Where it was deemed problematic, those candidates were dropped out of the study. The tables summarized the relevant number that were finally analysed. However, for the GPPAQ, the information from this instrument was first summarized in the manner in which its protocol requires prior to its analysis in Stata. This summary was done on its web-based application which yielded four categories of outcome that were subsequently analysed in the Stata statistical software. Continuous variables like age were categorised.

Risk factors were analysed and reported by proportions among the following groups; sex; rural and urban dwellers; age groupings; and disease status. A lot of attention was given to analysis of the strength of association and interaction of key predictors like obesity, physical activity and triglyceride levels. Odds ratios (ORs) were obtained as the measure of association between the risk factors and T2DM in the bivariate analysis. Adjusted ORs were obtained following the multivariate logistic regression analysis whiles controlling for individually significant covariates in each group of exposures. There was a simple score adopted for the REAP questionnaire in which we simply score from zero to three for each of the 27 main question items on the REAP tool. The total for the responses was calculated as the score for individual respondents. These results were categorized into four groups and analysed.

## Results

### Descriptive statistics

A total of 136 respondents participated in the study, consisting of 48 (35.29%) cases and 88 (64.71%) controls respectively. A total of 95 [39 (81.25%) cases and 56 (63.64%) controls] respondents underwent laboratory testing for fasting blood glucose and fasting blood lipid (total cholesterol, HDL cholesterol and triglycerides). By gender, there were more women (58.09%) than men (41.91%). The youngest subject was 35 years old and the oldest was 62 years old. The mean age of the respondents was 47.68 (sd = 6.98) years. Majority of the respondents were in the 40–49 years age group 63 (46.32%) of the total.

### Socio-demographic, anthropometric and physical activity factors

The place of residence of the respondents was grouped into urban and rural depending on the level of development of the area and whether it is a sub-district of the Ho Municipality. Majority (57.35%) of participants were grouped into places considered as urban as against 42.65% of rural dwellers. Interestingly, urban dwellers were in the majority (75%) among the cases, compared to 25% of rural dwellers. The bivariate analysis comparing the effect of the place of residence on risk of developing T2DM gave an OR of 3.29 (p < 0.0018) comparing urban dwellers to rural dwellers. This relationship continues to hold true when SES or body mass index (BMI) are controlled for individually (p < 0.05). Nevertheless, when physical activity level (GPPAQ) scores are controlled, the OR changes to 0.90 (p < 0.852).

Information obtained from the subjects on educational level, income level and type of work or employment status was used to derive a SES score for each participant. The scores were further grouped into three to represent low, middle and high strata of SES. Respondents in the middle and high strata of SES were compared to those in the low SES as a reference group, and the following results were obtained. Those in the middle SES had an OR of 2.97 (p < 0.007) while those in the high SES had an OR of 0.9 (p < 0. 929). After controlling for the effects of place of residence, GPPAQ score and BMI the OR for the middle SES increase to 5.03 (p < 0.003).

The GPPAQ which was employed to measure the physical activity level of the respondents typically gives four outcomes of activity levels. They are active, moderately active, moderately inactive and inactive. The active group of respondents was used as the reference group and subjects in the other groups were compared to them. For conveniences and ease of interpretation of results, the moderately inactive category was added to the inactive category and renamed inactive group. Respondents who were in the inactive group had an OR of 4.83 (p < 0.010). After controlling for BMI, place of residence and SES the OR changed to 7.30 (p < 0.011). BMI was calculated from the height and weight measurements and categorized into five different groups as summarized in Tables 1 and 2. Only the last category showed an OR of 6.6 (p < 0.028). After adjusting for the effect of physical activity level, SES and place of residence, the OR became 6.02 (p < 0.090).

**Table 1:** Background characteristics of respondents

Characteristics	Controls (n = 88)	Cases (n = 48)	Total (N = 136)
	n (%)	n (%)	n (%)
Age groups			
≤39	12 (13.64)	5 (10.42)	17 (12.5)
40–49	46 (52.27)	17 (35.42)	63 (46.32)
50–59	28 (31.82)	21 (43.75)	49 (36.03)
≥60	2 (2.27)	5 (10.42)	7 (5.15)
Gender			
Female	52 (59.09)	27 (56.25)	79 (58.09)
Male	36 (40.91)	21 (43.75)	57 (41.91)
Place of residence			
Rural	46 (52.27)	12 (25.00)	58 (42.65)
Urban	42 (47.73)	36 (75.00)	78 (57.35)
Educational level			
No formal schooling	11 (12.64)	3 (6.25)	14 (10.37)
Less than primary school	8 (9.20)	8 (16.67)	16 (11.85)
Completed primary school	10 (11.49)	4 (8.33)	14 (10.37)
Completed JHS	37 (42.53)	16 (33.33)	53 (39.26)
Completed SHS	9 (10.34)	4 (8.33)	13 (9.63)
Completed college/university	11 (12.64)	12 (25.00)	23 (17.04)
Post graduate degree	1 (1.15)	1 (2.08)	2 (1.48)
Type of work			
Government employee	14 (15.91)	14 (29.17)	28 (20.59)
Non-government employee	3 (3.41)	2 (4.17)	5 (3.68)
Self-employed	68 (77.27)	26 (54.17)	94 (69.12)
Homemaker	0 (0)	2 (4.17)	2 (1.47)
Retired	3 (3.41)	4 (8.33)	7 (5.15)
Alcohol consumption			
Daily	6 (11.11)	3 (10.34)	9 (10.84)
5–6 days per week	2 (3.70)	0 (0.00)	2 (2.41)
1–4 days per week	7 (12.96)	5 (17.24)	12 (14.46)
1–3 days per week	16 (29.63)	8 (27.59)	24 (28.92)
Less than once a month	23 (42.59)	13 (44.83)	36 (43.37)
Ever smoked			
No	82 (94.25)	44 (91.67)	126 (93.33)
Yes	5 (5.75)	4 (8.33)	9 (6.67)

**Table 2:** Crude odds ratios for bivariate analysis of the different groups of risk factors

Factors	Controls	Cases	Crude OR	CI (95%)	p value
	n (%)	n (%)			
*Socio-demographic, anthropometric and physical*					
Place of residence					
Rural	46 (52.27)	12 (25.00)	1		Ref.
Urban	42 (47.73)	36 (75.00)	3.29	1.51–7.14	0.0018
Socio-economic status					
Low	54 (70.13)	20 (46.51)	1		Ref.
Middle	20 (25.97)	22 (51.16)	2.97	1.34–6.57	0.0070
High	3 (3.90)	1 (2.33)	0.9	0.09–9.16	0.9290
GPPAQ score					
Active	16 (18.39)	4 (8.33)	1		Ref.
Moderately active	42 (48.28)	9 (18.75)	0.86	0.23–3.18	0.8180
Inactive group	29 (33.33)	35 (72.92)	4.83	1.45–16.05	0.0100
BMI (Kg/m^2^)					
<18.5	9 (10.23)	5 (10.42)	1		Ref.
18.5–24.9	43 (48.86)	11 (22.92)	0.46	0.13–1.65	0.2340
25.0–29.9	21 (23.86)	16 (33.33)	1.37	0.38–4.89	0.6260
30.0–34.9	12 (13.64)	5 (10.42)	0.75	0.17–3.40	0.7090
≥35	3 (3.41)	11 (22.92)	6.6	1.23–35.44	0.0280
*Dietary factors*					
Fruit consumption per week					
≤4	75 (86.21)	31 (64.58)	1		Ref.
≥5	12 (13.79)	17 (35.42)	3.43	1.47–8.01	0.0040
Servings of fruit per eating					
≥4	35 (40.23)	8 (16.67)	1		Ref.
2–3	38 (43.68)	28 (58.33)	3.22	1.30–8.01	0.0120
≤1	14 (16.09)	12 (25.00)	3.75	1.26–11.13	0.0170
REAP score					
≤30	15 (17.05)	2 (4.17)	1		Ref.
31–40	33 (37.50)	24 (50.00)	5.45	1.14–26.12	0.0340
41–50	35 (39.77)	17 (35.42)	3.64	0.75–17.78	0.1100
≥51	5(5.68)	5(10.42)	7.5	1.09–51.52	0.0400
*Biochemical factors*					
Total cholesterol (mmol/L)					
<5.2	40 (76.92)	22 (51.16)	1		Ref.
5.2–6.2	8 (15.38)	10 (23.26)	2.27	0.78–6.60	0.1310
≥6.3	4 (7.69)	11 (25.58)	5	1.42–17.58	0.0120
Triglyceride (mmol/L)					
<1.7	51 (98.08)	38 (88.37)	1		Ref.
≥1.7	1 (1.92)	5 (11.63)	6.71	1.42–17.58	0.0880
HDL cholesterol					
Best	29 (55.77)	18 (42.86)	1		Ref.
Better	6 (11.54)	3 (7.14)	0.81	0.18–3.63	0.7780
Poor	17 (32.69)	21 (50.00)	1.99	0.83–4.74	0.1210

The number of days within the week in which subjects consumed fruits was obtained and categorized into two. An OR of 3.43 (p < 0.004) was observed comparing those who consume fruits 5 or more days in a week to those who consume fruits up to 4 days in a week. Adjustment for other dietary components (servings of fruits per eating and REAP dietary score) yielded an OR of 4.08 (p < 0.003). Besides the frequency of fruit consumption, the servings of fruits consumption per eating was also obtained and categorized into three. Those who eat four or more servings of fruits; those who eat two to three servings of fruits; and those who eat one or less servings of fruits. Those who eat four or more servings were the reference group. The ORs were 3.22 (p < 0.012) and 3.75 (p < 0.017) 0.017 respectively. After adjusting for the effect of the REAP dietary score and frequency of fruit consumption per week on the development of T2DM, the ORs became 3.15 (p < 0.02) and 5.76 (p < 0.005) respectively. Subjects with REAP scores of 31–40; 41–50 and ≥51 compared with those having scores ≤30 had ORs of 6.09 (p < 0.035); 5.86 (p < 0.046) and 7.34 (p < 0.055) respectively after adjusting for fruit consumption.

The levels of total cholesterol were categorized as shown in Table 2 with the ≤5.2 mmol/L category being the reference group. Respondents with total cholesterol values 5.2–6.2 mmol/L and those ≥6.3 mmol/L had ORs of 2.27 (p < 0.131) and 5.00 (p < 0.012) respectively. After adjusting for the effect of BMI and HDL cholesterol, those in the 5.2–6.2 mmol/L group had an OR of 4.09 (p < 0.033) while those with ≥6.3 mmol/L cholesterol values had an OR 10.67 (p < 0.002). For triglycerides, the cut-off point was 1.7 mmol/L and those with fasting triglyceride levels of ≥1.7 mmol/L had an OR of 6.71 (p < 0.088). HDL cholesterol was grouped into three. The best group (<1.0 mmol/L for men and <1.3 mmol/L for women), better group (1–1.3 mmol/L for men and 1.3–1.5 mmol/L for women) and poor group (> 1.3 mmol/L for men and > 1.5 mmol/L for women). The reference group was the best group and the following ORs were obtained; 0.81 (p < 0.778) and 1.99 (p < 0.121) for the better and poor groups respectively. Interestingly the situation changes dramatically for the poor group of HDL cholesterol in the presence of total cholesterol. The OR then becomes 4.74 (p < 0.007).

## Discussions

The age range for participants in the study was from 35 to 65 years. There was no statistical difference in the mean age of the respondents comparing females to males. Also age as a continuous variable was significantly associated with the chances of developing T2DM. However, after categorization of age into four groups this association was completely lost. Again, controlling for age with physical activity levels showed no association. This suggests that age may be a confounder in this study (Table 3).

**Table 3:** Adjusted odds ratios of factors associated with T2DM by their groups

Factors	Crude OR	Adjusted OR	CI (95%)	p value	chi2
*Socio-demographic, anthropometric and physical*					40.51
Place of residence					
Rural	1	Ref.			
Urban	3.29	0.90	0.31–2.64	0.852	
Socio-economic status					
Low	1	Ref.			
Middle	2.97	5.03	1.71–14.74	0.003	
High	0.9	0.84	0.03–21.34	0.915	
GPPAQ score					
Active	1	Ref.			
Moderately active	0.86	0.98	0.21–4.57	0.978	
Inactive group	4.83	7.30	1.59–33.46	0.011	
BMI (Kg/m^2^)					
<18.5	1	Ref.			
18.5–24.9	0.46	0.64	0.13–3.23	0.588	
25.0–29.9	1.37	1.74	0.36–8.54	0.494	
30.0–34.9	0.75	0.57	0.08–3.83	0.563	
≥35	6.6	6.02	0.76–47.98	0.090	
*Dietary factors*					24.15
Fruit consumption per week					
≤4	1	Ref.			
≥5	3.43	4.08	1.59–10.4	0.003	
Serving of fruit per eating					
≥4	1	Ref.			
2–3	3.22	3.15	1.2–8.29	0.02	
≤1	3.75	5.76	1.69–19.6	0.005	
REAP score					
≤30	1	Ref.			
31–40	5.45	6.09	1.14–32.5	0.035	
41–50	3.64	5.86	1.03–33.1	0.046	
≥51	7.5	7.34	0.96–56.3	0.055	
*Biochemical factors*					16.1
Total cholesterol (mmol/L)					
<5.2	1	Ref.			
5.2–6.2	2.27	4.09	1.12–14.97	0.033	
≥6.3	5	10.67	2.41–47.23	0.002	
HDL cholestrol*					
Best	1	Ref.			
Better	0.81	2.03	0.38–10.94	0.41	
Poor	1.99	4.74	1.52–14.86	0.007	

* HDL cholesterol only becomes significant in the presence of total cholesterol values

Most of the respondents were self-employed with the second largest group being government employees. The mode of employment was not directly assessed in this study but was factored into the definition of SES for further assessment. It is common practice to combine elements of education, income level and occupation in defining SES as is apparent with some researchers [23, 24]. Only two (2) respondents currently smoke. For those who have ever smoked, we have a prevalence of 6.67%. This is slightly different from 9.7% that was obtained in the Ashanti Region in a cross-sectional survey of the region [25]. Nevertheless both studies affirm the low prevalence of smoking in Ghana.

The place of residences initially showed a strong association with the risk of developing T2DM with urban dwellers having about three (3) times the chances of developing T2DM compared to rural dwellers even after controlling for SES and BMI. However, this relationship does not hold true within strata of physical activity score (GPPAQ). After controlling for physical activity, the OR came to 0.90 (p < 0.852). This finding is in consonance with the results of some researchers in France who reported that rural dwellers are more likely to meet the recommended physical activity levels [26] than urban dwellers. Interestingly some other researchers in USA found the same evidence of greater physical activity levels in sub-urban dwellers when compared with inner city dwellers [27].

SES was found to be a significant determinant for developing T2DM. Interestingly the Middle social class was shown to be the worst affected. People in the Middle social class have more than five (5) times the chances of developing T2DM after the effects of physical activity, BMI and place of residence have been controlled for. This observation is rare. Many studies have reported an increase in the risk of developing T2DM among the low socio-economic class instead [28–30].

Physical inactivity is a significant determinants of T2DM. The inactive group of respondents have almost six (6) times the chances of developing T2DM compared with those who are active. In the consensus statement of the American diabetes association, the evidence of the protective property of physical activity was emphasized [31]. BMI of the respondents is apparently quiescent until the ≥35 kg/m^2^ where it suddenly rises with an OR of 6.60 (p < 0.028). After controlling for physical activity level, place of residence and SES however, the OR drops to 6.06 (p < 0.090). Similar trend of very large change in the predictability of T2DM by BMI was observed in a prospective cohort study involving 27,270 men [32]. However, for their work BMI of ≥35 kg/m^2^ was lumped together with ≥30 kg/m^2^. They however obtained a significant relative risk value of 7.9 [33].

The number of days per week that one consumes fruits had a positive relationship with the development of T2DM. Individuals who consumed fruits for five (5) or more days in the week had four (4) times the chances of developing T2DM compared to those who eat fruits on fewer days. This could be resultant from a change in life style among cases following a diagnosis of T2DM. The servings of fruits consumed however, had a negative correlation with T2DM. Respondents who consumed more servings of fruits were protected against T2DM. Consumption of one (1) or less servings of fruits increased one’s odds of developing T2DM to about six (6) times compared with the consumption of four or more servings per eating. The relationship between consumption of fruits and diabetes has often attracted contradicting evidence. Some researchers assert that increase intake of fruits significantly reduce the risk of diabetes [34]. The finding in this study agrees with this. Individuals diagnosed with T2DM are often educated about the benefits of taking fruits. This could make them take fruits more often than the controls.

The REAP dietary score showed to be positively associated with the development of T2DM. In general, beyond a score of 30, the chance of developing T2DM is increase by an average of about five (5) times. After adjustment for BMI and fruits consumption, the *p* value of the ≥51 group is slightly attenuated to 0.055 and that segment alone become insignificant.

The triglyceride levels were not found to be significantly associated with T2DM in this study. Of all the biochemical factors assessed, total cholesterol levels showed the highest ORs even after adjustment for BMI which is also an indirect measure of body fat. Individuals with total cholesterol levels in the ≥6.3 mmol/L range had over ten (10) times the chance of developing T2DM compared to individuals in the <5.2 mmol/L group. HDL cholesterol interestingly seemed not to be strongly associated with the development of T2DM. However, in the presence of total cholesterol HDL cholesterol suddenly becomes a very significant determinant of T2DM. This study asserts to lower HDL cholesterol and higher triglyceride levels among diabetics compared to non-diabetics.

Finally, reverse causation could have possibly affected the biochemical and dietary factors among old cases leveraging the differences in risk factors between cases and controls. This is because management of T2DM involves both dietary and pharmaceutical therapy both of which have the potentials to affect the dietary and biochemical factors measured in this study. The differences observed could have been possibly larger.

### Limitations of the study

Analysis on differences in the risk factors according to ethnic groupings could not be performed because majority of our participants were of one ethnic group (Ewes). Genetic predisposition by way of family history of diabetes mellitus was not assessed. A total of 136 candidates were recruited instead of the 137 candidates as required by the sample size calculation using the Fleiss formula/method in the EpiData 7 Software.

For majority of the cases, they were old T2DM patients so recall bias is a possibility. However for those cases who were newly diagnosed or diagnosed by the work of the research team, this is less likely. However there was no separate sub-group analysis between incident and prevalent cases to draw a conclusion on these aspects. This is because the incident cases were too few.

## Conclusions

In our study, individuals in the middle SES are at a greater risk of developing T2DM compared to others and this was found to be very significant. This finding underscore the need for researchers to evaluate the risk factors for NCDs in the local settings since the findings may turn out to be very different from what is known elsewhere.

This study indicated that physical activity level confounds the relationship between place of residence and development of T2DM. And that dwelling in rural areas in itself does not protect against T2DM but rather the physical activity elements in life involved in having to live in a rural setting is the key to protecting against T2DM among rural dwellers. Therefore a low physical activity level is a valid determinant of T2DM irrespective of BMI level, SES or place of residence. Again this may support the validity of the use of the GPPAQ in a Ghanaian setting. Eating large quantities/servings of fruits per seating provides protection against development of T2DM.

Promulgating policies aimed at providing work, revenue and or tax incentives for individuals in the middle SES who are willing to reside and work in rural areas or communities could help attract and retain these individuals to live and work in rural communities. Once in these communities they will be more physically active and have a higher likelihood of greater fruit consumption in their diets as well. Thus reducing the risk factors and incidence of T2DM.

## Abbreviations

BMI: body mass index
DM: diabetes mellitus
GPPAQ: general practice physical activity questionnaire
LADA: latent autoimmune diabetes of adults
MODY: maturity onset diabetes of the young
NCD: non-communicable diseases
OPD: out-patient department
REAP: rapid eating and activity assessment for patients
SES: socio-economic status
T1DM: type 1 diabetes mellitus
T2DM: type 2 diabetes mellitus
WHO STEPS: WHO STEPwise approach to surveillance d tools were incorporated
